# Identifying latent dynamic components in biological systems

**DOI:** 10.1049/iet-syb.2014.0013

**Published:** 2015-10-01

**Authors:** Ivan Kondofersky, Christiane Fuchs, Fabian J. Theis

**Affiliations:** ^1^ Institute of Computational Biology German Research Center for Environmental Health Helmholtz Zentrum München Ingolstädter Landstr. 1 85764 Neuherberg Germany; ^2^ Center for Mathematics Chair of Mathematical Modeling of Biological Systems Technische Universität München Boltzmannstr. 3 85748 Garching Germany

**Keywords:** biology computing, RNA, splines (mathematics), maximum likelihood estimation, approximation theory, biochemistry, latent dynamic components, biological systems, computational system biology, regulatory models, multivariate readouts, biological applications, external regulations, real‐world examples, microRNA, metabolic fluxes, latent dynamic variables, variable time course, two‐step procedure, spline approximation, maximum‐likelihood estimation, model selection, signalling pathway model, real data, incomplete network structures

## Abstract

In computational systems biology, the general aim is to derive regulatory models from multivariate readouts, thereby generating predictions for novel experiments. In the past, many such models have been formulated for different biological applications. The authors consider the scenario where a given model fails to predict a set of observations with acceptable accuracy and ask the question whether this is because of the model lacking important external regulations. Real‐world examples for such entities range from microRNAs to metabolic fluxes. To improve the prediction, they propose an algorithm to systematically extend the network by an additional latent dynamic variable which has an exogenous effect on the considered network. This variable's time course and influence on the other species is estimated in a two‐step procedure involving spline approximation, maximum‐likelihood estimation and model selection. Simulation studies show that such a hidden influence can successfully be inferred. The method is also applied to a signalling pathway model where they analyse real data and obtain promising results. Furthermore, the technique can be employed to detect incomplete network structures.

## 1 Introduction

A central objective in computational systems biology is to identify components of biological system networks and their relation to one another [1, 2]. For the prediction of time‐resolved, dynamical network behaviour, mathematical models are employed that typically involve several unknown parameters in addition to the network components. A popular modelling approach for time‐resolved measurements is given by ordinary differential equations (ODEs) that represent the dynamics of and dependencies between the components of the network. The parameters describing the dynamics in an ODE must be inferred statistically, and in the case of several competing network models, the most appropriate model can be chosen by model selection methods. Hence, one deals with a mathematical modelling problem and a statistical estimation problem, simultaneously [3].

In such an analysis, ODEs directly arise from the network topology, that is, the modeller specifies the components of the network and possible interactions. In many applications, the key elements of the dynamics of interest have been previously determined in various studies and are well‐known from the literature. It is possible, however, that some interaction partners or connections remain unspecified. For example, in addition to transcription factors modulating gene regulation, strong evidence indicates that microRNAs play an important role in transcription and translation processes [4]. Translation can also be influenced by external stimuli like drugs [5, 6, 7]. Consequently, a mathematical model may be insufficient to explain the dynamics of interest, that is, discrepancies with the measured data which are not simply because of the measurement error may be evident even with the best model fit.

A promising way of addressing such discrepancies is given by employing additional network components to extend the proposed model. Our main focus in this paper is a systematic model extension. A substantial amount of work has been conducted in the past years in this field.

In [8], additional links in undirected graphs are identified using Gaussian graphical models. These links represent model extensions and are systematically identified using an *l*_1_‐penalised likelihood. However, the proposed algorithm is not applicable to dynamical data.

Gao *et al.* [9] and Honkela *et al.* [10] also consider a model extension, this time for dynamical data. Similar to the approach to be presented here, they describe their models in terms of ODEs with a latent variable. Using Gaussian processes, they infer the time course of this variable and predict its behaviour. However, they do not model entire networks, which may possibly involve numerous links between components, but rather focus only on transcription and translation of single genes and on analytical solutions of the specific ODE models.

Furthermore, model extension by latent variables is utilised in the context of latent confounder modelling [11, 12]. Here, the most frequently used method is structural equation modelling (SEM) [13, 14]. SEM allows the identification of multiple latent variables and their relationship with observed variables by exploiting the data covariance structure. SEM is mainly formulated for single time points, and an extension to dynamical data is quite limited and often not possible.

In the present study, we address the problem of poor model quality in dynamical models by considering the effect of hidden influences on the network. We do not assume a functional form for the putative time courses of such hidden processes, but flexibly estimate their dynamics and interaction strengths. Wherever a hidden influence is observed that substantially improves the model's ability to represent the data, we attempt to provide a biological meaning with the help of experimental collaborators. Thus, we can guide the design of additional experiments in a detailed manner by providing a quantification of the hidden time courses as well as relative interaction rates between the hidden components and the existing network.

This paper is organised as follows. Section 2 presents the ODE models considered and the means by which a hidden influence is included therein as well as a schematic representation of the developed method. The hidden component and the model parameters are statistically estimated in a two‐step procedure, as described in Section 3. Furthermore, this section also discusses parameter uncertainty and explains how the most appropriate model among several competing ones can be chosen with model selection methods. The proposed method is applicable to Lipschitz continuous ODE models, for example, gene regulation models or signal transduction models. Section 4 applies the developed technique to different scenarios and to a real data example – the JAK2‐STAT5 signalling pathway. Section 5 concludes this paper and discusses the strengths and limitations of the proposed method.

## 2 Approach

In this section, we highlight the main ideas of the present study. Systematic network extension is illustrated by considering a small motif example. Next, we generalise this extension to networks of size *N*.

**Fig. 1:**
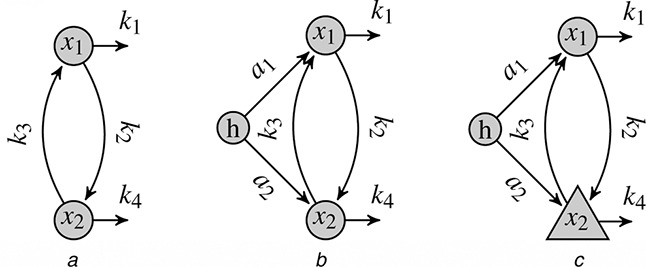
Example network motifs (circles: observed and hidden components; triangle: unobserved or indirectly observed component) *a* Motif without hidden components, that is, all components are observed *b* Motif with a single hidden component (*h*), where all other components are observed *c* Motif with a single hidden component (*h*) and partially observed components Partially observed networks are discussed in Section 3.5

Consider a simple motif like the one presented in Fig. 1.

We use the schematic representation of small network motifs shown in Fig. 1 to illustrate our method as follows. Fig. 1*a* shows a simple network motif comprising two components *x*_1_ and *x*_2_, which influence each other, as indicated by the corresponding arrows. With the proposed method, we estimate a hidden component *h*, shown in Fig. 1*b*, which may substantially contribute to the network dynamics, but was not previously considered. Thus, we call *h* a ‘hidden influence.’ Fig. 1*c* stresses that not all components must be observed.

ODEs are the most prevalent choice for describing such network dynamics [15]. We assume these to be Lipschitz continuous; thus, the existence and uniqueness of an ODE solution are guaranteed. For motif A in Fig. 1, the corresponding equation is

(1) $${\dot{x}}_{i}(t)=\psi_{i}(k,x(t))$$

with parameter vectors ***k*** = (*k*_1_, …, *k_L_*)^T^, *k_l_ ∈ R*_≥0_, non‐negative state vector ***x*** (*t*) = (*x*_1_(*t*), …, *x_N_* (*t*))^T^, *x_i_* (*t*) *∈ R*_≥0_, derivatives with respect to time ${\dot{x}}_{i}(t)$, possibly non‐linear functions $\psi_{i}:R_{\geq 0}^{p}\times R_{\geq 0}^{N}\to R$ and suitable initial values *x_i_* (*t*_0_), where *t* ≥ *t*_0_ represents the time. The functions *ψ_i_* generate the network structure and may include several combinations of the state variables ***x*** (*t*) such as linear combinations, Michaelis–Menten kinetics, complex formation and others. The connection between the state variables is described by the parameters ***k***.

The components *x_i_* (*t*) may be observed or unobserved. In addition to the motif in Fig. 1*a*, we now assume a time‐varying hidden component *h*(*t*) that acts linearly on ${\dot{x}}_{i}(t)$, as shown in Fig. 1*b*. The system of differential equations then changes to

(2) $${\dot{x}}_{i}(t)=\psi_{i}(k,x(t))+a_{i}h(t)$$

with weights ***a*** = (*a*_1_, …, *a_N_*)^T^, *a_i_ ∈ R*. Positive weights *a_i_* in this context represent activation of the *i*‐th component, whereas a negative value of *a_i_* implies inhibition. A similar model was considered, e.g. in [16]. Other than for *x_i_* (*t*), we do not assume any parametric structure for the hidden component. The time course of *h*(*t*) cannot be observed directly.

Six elements determine the model: the components *x_i_* and their time derivatives ${\dot{x}}_{i}$, the parameter vectors ***k*** and ***a***, the dependency describing functions *ψ_i_* and the hidden influence *h*. We will extend established models from the literature by adding hidden components and applying our estimation method described in Section 3. For reasons of simplicity, we assume that the reaction rates ***k*** and dependency functions *ψ_i_* are known. Both assumptions can also be relaxed, as is demonstrated in Section 4.2 where we additionally estimate ***k*** and recover a missing feedback, thus altering the network structure.

**Fig. 2:**
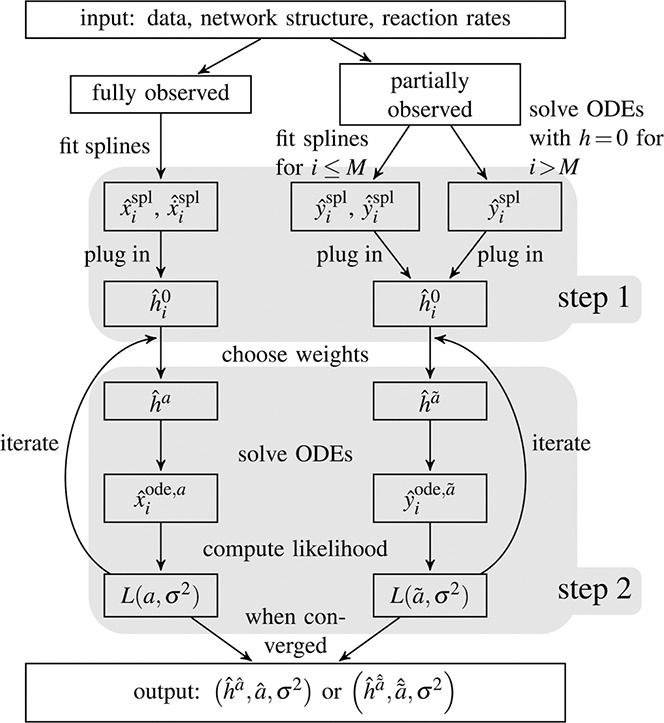
Flowchart illustrating the details of the developed method Method involves estimating hidden components according to two major steps (indicated by grey boxes). We distinguish between fully and partially observed networks. If at least one network component is unobserved, the procedure requires a preliminary step where the system of ODEs is solved without considering a hidden component. As a next step in both scenarios, penalisation splines are fitted to the measurements using cross‐validation for the estimation of the smoothing parameter. Finally, a maximum‐likelihood loop is performed until convergence to estimate the time course of the hidden component and its interaction weights as well as the noise parameter *σ* ^2^

The objective of our study is to estimate *a_i_* and *h*(*t*), and examine if they improve the ability of the model to represent the data; this also requires the estimation of ***x*** and $\dot{x}$. In our analysis, we exploit the following connection between *h* and all other components

(3) $$h(t)=\frac{{\dot{x}}_{i}(t)-\psi_{i}(k,x(t))}{a_{i}}$$

for all *t* and *i* with *a_i_ ≠* 0. The hidden influence can then be estimated according to two major steps as follows. First, we fit penalisation splines to the measurements of ***x***. This allows a direct computation of the time derivatives $\dot{x}$ such that the right‐hand side of (3) is known up to a scaling factor *a_i_*. These factors are then estimated via likelihood maximisation, utilising the differential equation structure. A flowchart that illustrates the details of the developed method is shown in Fig. 2.

## 3 Methods

This section describes the above‐mentioned two‐step procedure for the estimation of the hidden influence *h*. As a basis, we assume observations $x_{i}^{\text{obs}}(t_{j})$ of all components *x*_1_, …, *x_N_* at discrete time points *t*_0_, …, *t_n_*. The first step is presented in Section 3.1, where we use spline functions to approximate the time courses of *x*_1_, …, *x_N_* and their derivatives. In a second step, we define a noise model for the data and estimate the weights *a_i_* using likelihood maximisation in Section 3.2. In Section 3.3, we discuss uncertainty and the fit quality of the parameters of interest. In Section 3.4, we perform model selection on the considered networks. Finally, in Section 3.5, we extend the estimation methods to the case of partially observed networks.

### 3.1 Spline estimation for observed time courses and their derivatives

Splines are a convenient way to approximate the time course of a series of measurements in a functional form and are successfully used to model time‐resolved, biological data, for example, [17]. They typically arise as a linear combination $\sum_{k}\beta_{k}\phi_{k}(t)$ of known basis functions *ϕ*_1_, …, *ϕ_K_* with corresponding coefficients *β*_1_, …, *β_K_ ∈ R*, as described, for example, in [18]. These coefficients are chosen such that the differences between the measured data and the corresponding function values are minimised. The wiggliness of the spline depends on, in addition to other settings, the dimension *K* of the basis spanning the functional space. A large *K* can result in overfitting in the sense of exaggerated data faithfulness. In this case, the spline will perfectly resemble the presently observed data but poorly predict future measurements. A low value for *K*, on the other hand, can lead to an oversimplified approximation of the true dynamics. To achieve a good trade‐off between these two extremes, we use penalisation splines where the wiggliness of the spline is controlled by an additional smoothing parameter *λ* > 0 [19]. Applied to the model given in (2), for a given *λ_i_*, the basis coefficients *β*_1*i*_, …, *β_Ki_* are chosen such that the following term is minimised [20]

(4) $$\begin{array}{r} {\left(\;x_{i}^{\text{obs}}-\sum_{k=1}^{K}\beta_{ki}\phi_{k}\;\right)}^{\;\;T}\left(\;x_{i}^{\text{obs}}-\sum_{k=1}^{K}\beta_{ki}\phi_{k}\;\right)+\lambda_{i}\int_{t_{0}}^{t_{n}}{\left(\sum_{k=1}^{K}\beta_{ki}{\ddot{\phi }}_{k}(s)\;\right)}^{\;\;2}\;ds \end{array}$$

In this notation, $x_{i}^{\text{obs}}=(x_{i}^{\text{obs}}(t_{0}),\ldots ,x_{i}^{\text{obs}}(t_{n})){\;}^{T}$ is the vector of the measured data, and the basis functions evaluated at the observation times are denoted by *ϕ_k_* = (*ϕ_k_* (*t*_0_), …, *ϕ_k_* (*t_n_*))^T^. As the index *i* in *λ_i_* suggests, a penalisation parameter is chosen for each component separately.

In this study, we choose a sufficiently large *K* and estimate *λ_i_* using leave‐one‐out cross‐validation. Hereby, splines are estimated with *n* observations and one observation is left out. The excluded observation is subsequently compared to the spline approximation at the same time point. This comparison is repeated for all *n* + 1 observations and different values of *λ_i_*. Finally, this procedure leads to an optimal *λ_i_* for which the spline approximation is close to the data without overfitting it. Alternatively, one could use bootstrap techniques or more generalised cross‐validation criteria for this purpose, as discussed in [20].

Minimisation of (4) yields optimal coefficients ${\hat{\beta }}_{ki}$, and consequently an approximation for the time course of the observed components

(5) $${\hat{x}}_{i}^{\text{spl}}(t):=\sum_{k=1}^{K}{\hat{\beta }}_{ki}\phi_{k}(t)$$

and their time derivatives

(6) $${\hat{\dot{x}}}_{i}^{\text{spl}}(t):=\frac{\partial {\hat{x}}_{i}^{\text{spl}}(t)}{\partial t}=\sum_{k=1}^{K}{\hat{\beta }}_{ki}{\dot{\phi }}_{k}(t)$$

The estimation of the time derivatives given by (6) plays an equally important role as the estimation of the splines given by (5) for the final estimation of the time course of the hidden component given by (3). Thus, particularly for the analysis of very noisy data, additional smoothing techniques, such as a higher penalisation order in (4), may be considered. Furthermore, the shape of the estimated spline is not only determined by the parameters *K* and *λ_i_* but also by the type of basis functions used. Common types of spline functions are B‐splines, Fourier splines (applicable to, e.g. for periodic data), polynomial splines, among others [20]. We implemented our method to include all these possibilities. The results presented in Section 4 are based on cubic B‐splines defined on an equally spaced time grid. These functions are twice continuously differentiable such that the penalisation term in (4) is well‐defined [20]. In practice, however, the integral is approximated through finite differences [20]. For that reason, we only require the basis functions to be at least once differentiable so that (6) is well‐defined.

With the approximations given by (5) and (6), we can now estimate the numerator in (3)

(7) $${\hat{h}}_{i}^{0}(t_{j})={\hat{\dot{x}}}_{i}^{\text{spl}}(t_{j})-\psi_{i}(k,{\hat{x}}^{\text{spl}}(t_{j}))$$

For the estimation of the denominator in (3), we apply a likelihood approach, as described in the following section.

### 3.2 Maximum‐likelihood estimation

Given a weight vector ***a***, the hidden influence can be approximated as

(8) $${\hat{h}}^{a}(t_{j})=\frac{1}{N^{a}}\sum_{\left\{i:a_{i}\neq 0\right\}}\frac{{\hat{h}}_{i}^{0}(t_{j})}{a_{i}}$$

where *N**^a^*** is the number of non‐zero weights *a_i_*. The case ***a*** = **0** can be excluded without loss of generality because it indicates the absence of a hidden influence extending the network. This approximation of *h* will later be plugged into (2) where it is multiplied with *a_i_*. If ${\hat{h}}_{i}^{0}$ is the true numerator of (3), the time courses ${\hat{h}}_{i}^{0}$ on the right‐hand side will all be identical up to a scaling factor. However, because it is an approximation there will be differences between them in practice. For this reason we consider the pointwise weighted average in (8), which presents a natural choice of a summary statistic. If it holds that the single estimates ${\hat{h}}_{i}^{0}(t_{j})$ strongly differ from each other, then the weighted average ${\hat{h}}^{a}(t_{j})$ will be inaccurate, and this in turn will be reflected in the likelihood function and the corresponding information criterion that we later formulate in (13) and (16), respectively, thus leading to the rejection of the proposed model.

Plugging in *h* into (2) and multiplying it by *a_i_* introduces a non‐identifiability. Because of

(9) $$a_{i}{\hat{h}}^{a}=(\xi a_{i})\left(\frac{{\hat{h}}^{a}}{\xi }\right)$$

for any *ξ ≠* 0, the weights *a_i_* are non‐identifiable. For this reason, we restrict ***a*** to $\sum_{i}|a_{i}|=1$. In the special case of a network consisting of only one component *x*_1_, we only estimate the interaction direction of the hidden influence, that is, *a ∈* { − 1, 1}.

In most biological applications, the data contains noise of different origins, such as measurement noise or technical noise [21, 22]. The most common assumption is that measurement errors are independent and normally distributed with mean zero and constant standard deviation *σ* > 0

(10) $$x_{i}^{\text{obs}}(t_{j})=x_{i}(t_{j})+\varepsilon_{ij},\;\varepsilon_{ij}\overset{\text{iid}}{∼}N(0,\sigma^{2})$$

In applications, *x_i_* (*t_j_*) often has a positive domain, and in this case, (10) might be ill‐defined. Note that we do not restrict our methods to only this type of noise. In Appendix 1, we also specifically derive all the equations given in this section for log‐normally distributed multiplicative noise. The distribution of *ε_ij_* immediately propagates to the measurements

(11) $$x_{i}^{\text{obs}}(t_{j})|x_{i}(t_{j})\overset{\text{iid}}{∼}N\left(x_{i}(t_{j}),\sigma^{2}\right)$$

While the true time course *x_i_* (*t*) is unknown, it has already been approximated in (5). This approximation, however, does not contain any information about ***a***, which we seek to estimate in the following. Hence, we introduce another approximation for *x_i_* (*t*), this time exploiting the ODE structure given in (2): for a given ***a***, we plug in ${\hat{h}}^{a}$ from (8) into the ODE given in (2) and solve the differential equations either analytically or numerically, as described, for example, in [23]. This yields ${\hat{x}}_{i}^{\text{ode},a}(t_{j})$ and leads to the approximate distribution

(12) $$x_{i}^{\text{obs}}(t_{j})|{\hat{x}}_{i}^{\text{ode},a}(t_{j})\overset{\text{iid}}{∼}N({\hat{x}}_{i}^{\text{ode},a}(t_{j}),\sigma^{2})$$

Overall, we arrive at the conditional likelihood function

(13) $$L(a,\sigma^{2})=\prod_{i=1}^{N}\prod_{\;j=0}^{n}f_{a,\sigma^{2}}(x_{i}^{\text{obs}}(t_{j}))$$

where $f_{a,\sigma^{2}}$ is the probability density function corresponding to the chosen error specification.

In addition, a conditional estimate for *σ* ^2^ can be derived analytically

(14) $${\hat{\sigma }}_{\text{ML}}^{2}=\frac{1}{N(n+1)}\sum_{i=1}^{N}\sum_{\;j=0}^{n}{\left(x_{i}^{\text{obs}}(t_{j})-{\hat{x}}_{i}^{\text{ode},a}(t_{j})\right)}^{2}$$

The parameters ***a*** and *σ* ^2^ are jointly estimated using (14) and a numerical optimisation of (13). In addition, unknown initial conditions ***x*** (*t*_0_) are treated as unknown parameters and are equivalently estimated.

### 3.3 Parameter uncertainty

We further explore our likelihood approach with respect to parameter uncertainty. The overall estimation performance of the unknown parameters *σ* ^2^ and ***a*** can be analysed by computing the Cramer‐Rao lower bound (CRLB) [24, 25] which is defined as the inverse expected Fisher information matrix. This theoretical value describes a lower bound for the mean squared error (MSE) of a given parameter. To that end, we look at the diagonal elements of the expected Fisher information matrix, which, in the case of a normally distributed error, have the following form

(15) $$I_{k}(a,\sigma^{2})=\left\{\begin{array}{cc} \frac{1}{\sigma^{2}}\sum_{i}\sum_{j}{\left(\frac{\partial }{\partial a_{k}}{\hat{x}}_{i}^{\text{ode},a}(t_{j})\right)}^{2} & k\leq N\\ N(n+1)/\left(2\sigma^{4}\right) & k=N+1 \end{array}\right.$$

In practice, we solve the ODEs numerically. Here, a sophisticated ODE solver, such as the Runge‐Kutta fourth order method [26] can be employed to produce accurate estimates. However, an analytical derivation of the CRLB for such a method becomes very complex because of the complicated recursive formulation of the ODE solution. For exemplary purposes, we outline a derivation for a specific small example using the Euler method in Appendix 2.

Large values on the diagonal of the expected Fisher information matrix represent parameters with a small CRLB. These parameters can be estimated accurately with an (asymptotically) efficient estimator. For the parameters *a_l_*, the respective *l*th diagonal element increases if

- *σ* ^2^ is small, that is, the data are subject to a small amount of noise,
- $(\left(\partial /\partial a_{k}){\hat{x}}_{i}^{\text{ode},a}(t_{j}\right)){\;}^{\;\;2}$ is large, that is, the ODE solution is sensitive to changes in the parameter *a_k_* and
- *n* and/or *N* are large, that is, the data arise from a large number of time points and different (observed) species.

For the parameter *σ* ^2^, we look at the (*N* + 1)th diagonal element of (15), which increases if

- *σ* ^2^ is small, that is, the data are subject to a small amount of noise and
- *n* and/or *N* are large, that is, the data arise from a large number of time points and different (observed) species.

We can conclude that, as expected, the estimation accuracy will suffer if we apply our method to small networks, few observations, conditions indicative of a weak influence of the hidden component and large noise. As indicated in Section 3.2, we estimate the parameters with a maximum‐likelihood approach. The estimation is asymptotically efficient [27]; thus, the CRLB is asymptotically achieved. However, the approximation of the time courses using splines as described in Section 3.1, introduces additional uncertainty. In Appendix 2, we examine this loss of accuracy for a given showcase network and various parameter combinations, thereby concluding that our method produces estimates that are very close to the CRLB.

### 3.4 Model selection

The vector ***a*** controls the interaction strength between the hidden influence *h* and the network components *x_i_*. If a weight *a_i_* is estimated to be close to zero, it will have a negligible effect on the network and will probably improve the model fit only slightly. In such a case, one may ask whether the inclusion of this parameter *a_i_* is worth the involved estimation effort or whether one should simply set this component equal to zero, thus reducing the complexity of the model.

To quantify the trade‐off between improved model fitting and increased model complexity, we consider the Akaike information criterion (AIC) and the Bayesian information criterion (BIC), which are established model choice rules [28, 29]

(16) $$\begin{array}{rl} \text{AIC}(\hat{\theta }) & =-2\log(L(\hat{\theta }))+2\dim(\hat{\theta })\\ \text{BIC}(\hat{\theta }) & =-2\log(L(\hat{\theta }))+\log\left((n+1)N\right)\dim(\hat{\theta }) \end{array}$$

In these equations, $\hat{\theta }$ denotes a vector containing all parameter estimates, $L(\hat{\theta })$ is the likelihood function (13) evaluated at $\hat{\theta }$ and $\dim(\hat{\theta })$ is the number of estimated parameters. The AIC and BIC weigh the accuracy of the fit (measured by the first summand) against the complexity of the model (measured by the second summand). One then chooses the model with the smallest AIC or BIC.

To consider the complexity of the overall estimation procedure, the vector ***θ*** can be chosen to include all unknowns determined in our two‐step approach, that is, all *λ_i_*, *β_ik_* and *σ* ^2^. In our considerations, however, the number of variables is constant apart from the number of non‐zeros *a_i_*. Hence, we can replace $\dim(\hat{\theta })$ by *N**^a^*** as defined in (8), to compare different models.

The models that we are considering with our method are all of a nested type. The special case of ***a*** = **0** is the null model and is nested within all other models with arbitrary ***a***. Regarding the decision of which values *a_i_* to set equal to zero, we follow three conventional variable selection methods: best subset selection, forward stepwise selection and backward stepwise selection. These and additional model selection methods are discussed in [30]. In the best subset selection, the AIC or the BIC is computed for all possible models, and the model with the best score is chosen. This approach is computationally expensive but guarantees to find the best model. The latter two methods are greedy algorithms that compute the AIC/BIC only for a fraction of possible models, which exploit the nested structure, thus saving computational time while yielding satisfactory results in practice.

In the forward stepwise selection, we begin with the model given in (2), which contains no interactions between the hidden influence and the network components, that is, all *a_i_* equal zero. In the second step, *N* models are estimated, where, for each of the models a different element of ***a*** is non‐zero while the others are held equal to zero. If the best model outperforms the selected model from the previous step, this model is accepted, and in the subsequent step, another component of ***a*** is set to a non‐zero value. This step is repeated until no increase in model performance is achieved with a more complicated model. Once a component *a_i_* is chosen to be non‐zero, it will remain non‐zero in all subsequent steps.

Backward stepwise selection is an analogy of the forward stepwise selection wherein the initial model selected is the most complicated model for which all interactions between the hidden and the other components are estimated. In each subsequent step, a single entry of ***a*** is fixed to zero until no lower value of AIC/BIC is achieved.

In Section 4, we employ the BIC for model choice on synthetic and real data because this criterion penalises the model complexity more than the AIC.

### 3.5 Partially observed network components

In the estimation procedure discussed in Sections 3.1 and 3.2, we assumed that the components *x_i_* were ‘directly’ observed and that ‘all’ of them were observed. In this section, we now consider the case where the observed time courses are affine linear transformations *y*_1_, …, *y_M_* of *x*_1_, …, *x_N_* and the number *M* of observed time courses is smaller than the total number of network components *N*. The flowchart shown in Fig. 2 illustrates the single steps of the estimation procedure.

As an example for non‐direct observations, consider the motif depicted in Fig. 1*c*, which is assumed to follow exactly the same dynamics as that in Fig. 1*b* and can therefore be described in terms of the ODEs given in (2). Suppose that one can now only measure time courses of the observation functions *y*_1_(*t*) = *x*_1_(*t*) and *y*_2_(*t*) = *bx*_2_(*t*) + *c* for scalars *b ≠* 0 and *c*. The ODEs given in (2) can then be translated to

(17) $${\dot{y}}_{m}(t)=\eta_{m}(\kappa ,y(t))+{\tilde{a}}_{m}h(t)$$

with appropriate *η_m_*, ${\tilde{a}}_{m}$ depending on ***a***, *b* and *c* and *κ* being the collection of the interaction rates ***k*** and transformation parameters *b* and *c*. As the observation functions ***y*** are affine linear transformations of the network components ***x***, we can extract the hidden influence following (7) and (8)

(18) $${\hat{h}}^{\tilde{a}}(t_{j})=\frac{1}{N^{\tilde{a}}}\sum_{\left\{i:{\tilde{a}}_{i}\neq 0\right\}}\frac{{\hat{\dot{y}}}_{i}^{\text{spl}}(t_{j})-\eta_{i}(\kappa ,{\hat{y}}^{\text{spl}}(t_{j}))}{{\tilde{a}}_{i}}$$

Note that, for non‐linear observation functions, we cannot directly apply our method possibly because of, for example, quadratic or higher order terms of *h*(*t*) in (17); however, in the above case, one can proceed in a manner analogous to that given in Sections 3.1 and 3.2 for the estimation of *h* and $\tilde{a}$ if both *y*_1_ and *y*_2_ are observed.

As an example of partial observation, we can assume that only *y*_1_ is observed. As the two‐dimensional (2D) ODE system given in (1) contains no redundant equation, the dynamics of interest are fully described by a network of only two components. Hence, in addition to the observed variable *y*_1_, we include one latent component (LC) *y*_2_ in our analysis, for example, *y*_2_ = *x*_2_ or *y*_2_ = *bx*_2_ + *c*, as discussed above. More generally, we consider a network with observed components *y*_1_, …, *y_M_* and unobserved components *y_M_*_+1_, …, *y_N_*. The estimation of a hidden influence and its weights changes slightly as opposed to the fully‐observed case because there is no spline approximation possible for the time courses of *y_M_*_+1_, …, *y_N_*.

In this case, we approximate *y*_1_, …, *y_M_* and their derivatives as before [see (5) and (6)]. Furthermore, we approximate *y_M_*_+1_, …, *y_N_* by their solutions of the *N*‐dimensional ODE system given by (17) with *h* ≡ 0. For simplicity, we denote these approximations by ${\hat{y}}_{i}^{\text{spl}}$ for all *i*, although there are no splines involved for *i* > *M*. The starting values *y_i_* (*t*_0_) are treated as additional unknown parameters. Owing to the ODE‐based derivation of ${\hat{y}}_{i}^{\text{spl}}$ for the latent variables, estimation of the corresponding ${\tilde{a}}_{i}$ is not feasible in the first step of the estimation procedure. Hence, we restrict the components of $\tilde{a}$ to be zero for *i* > *M*. For a given weight vector, the hidden influence is estimated through (18). In the second step, the likelihood function results as in (13) as a product over all observed components (*i ∈* {1, …, *M*}) and observation times (*j ∈* {0, …, *n*}). Maximisation of the likelihood function yields estimates for *h* and $\tilde{a}$ for all *i ∈* {1, …, *N*}.

## 4 Results

In this section, we demonstrate several different applications of our method. In Section 4.1, the prediction of the time course of a hidden component is evaluated. In Section 4.2, we present our method as a tool that guides the reconstruction of a previously misspecified network. Finally, in Section 4.3, we identify a LC in the JAK2‐STAT5 signalling pathway using real‐world data.

### 4.1 Method performance evaluated with synthetic data

To evaluate the performance of our method, we conduct several simulation studies. All test runs are performed with the statistical software R [31]. We examine the robustness of our method by varying the noise intensity of the simulated data. In addition, we evaluate networks of different sizes and study the dependence of the results on the number of unobserved components.

The parameters ***k*** and ***a*** are chosen at random for each simulation run, and conditioned on these, we generate artificial data at 30 equally spaced time points. We use log‐normal noise (see Appendix 1), and the three noise levels that we consider are low (*σ* = 0.01), medium (*σ* = 0.1) and high (*σ* = 0.3). In the simulation, we allow only linear interactions between the network components which, indicates that the structure of the ODEs can be summarised as

(19) $${\dot{x}}_{i}(t)=\sum_{u=1}^{N}\left(k_{iu}x_{u}(t)-k_{ui}x_{i}(t)\right)+a_{i}h(t)$$

with uniformly distributed *k_iu_* in [0, 1] for describing the reaction strength between the *i*th and *u*th components and uniformly distributed *a_i_* in [− 1, 1].

We use the same hidden influence for each simulation run, thus producing comparable results. After application of our estimation procedure, the resulting fit quality is measured by

(20) $$s=\frac{1}{n+1}\sum_{\;j=0}^{n}|\hat{h}(t_{j})-h(t_{j})|$$

**Fig. 3:**
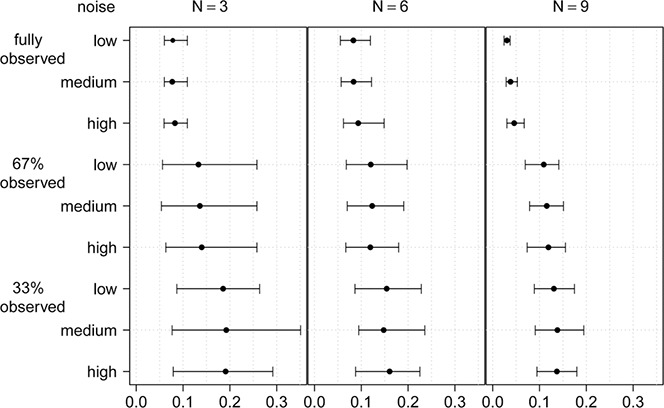
Description of the simulation studies used to evaluate the performance of our method For 27 different combinations of network size (3, 6 and 9 components), noise intensity (‘low’ ≡ *σ* = 0.01, ‘medium’ ≡ *σ* = 0.1 and ‘high’ ≡ *σ* = 0.3) and ratio of observed to unobserved components (100% observed, 67% observed and 33% observed), 100 different simulated networks are created and the mean as well as the 5 and 95% quantiles of the error measurement in (20) are displayed for each combination. All interaction rates between components are chosen randomly. It holds that, the smaller the value of *s*, the better the estimated time course

We estimate rates ***a*** with the forward selection technique. As illustrated in Fig. 3, results of 100 simulations indicate that a smaller network size and a smaller fraction of observed components lead to increasingly poor model fitting performance.

Only small differences are observed between low and high noise intensities, indicating that our method can accommodate a high degree of noise while extracting the relevant information from the data. In addition, it appears that the network size plays only a minor role with regard to the estimation quality of our method because the scores for larger networks decrease only slightly.

**Fig. 4:**
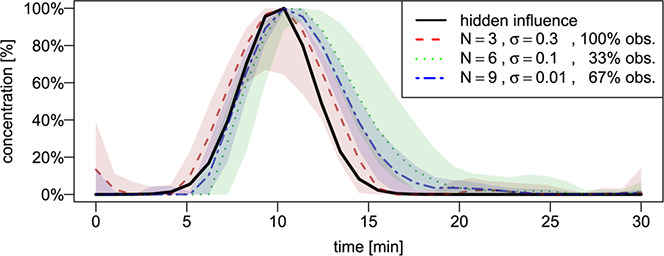
Time course of the hidden influence Hidden influence used in the simulations (solid line) and the mean and 5 and 95% pointwise quantile courses of three exemplary simulation scenarios with different parameters defined as follows: a fully observed network of size 3 with high noise intensity (dashed); a partially observed network (33%) of size 6 with medium noise intensity (dotted); a partially observed network (67%) of size 9 with low noise intensity (dashed‐dotted). Mean and confidence intervals are based on 100 estimates $\hat{h}(t)$

Our approach also yields estimates of the time course of the hidden component, which we can compare with the true hidden component used to generate the data. Fig. 4 shows the mean and 5 and 95% pointwise quantile time courses of three exemplary simulation scenarios. The shape of the hidden influence is reproduced satisfactorily, albeit differently. For a network comprising three components and a high degree of noise, the estimates produce additional fluctuations that are not present in the true time course and the confidence intervals are very broad. For a larger network size (6 or 9 components), the estimates become more stable and recover the peak of the true time course; however, the second part of the peak is slightly overestimated because of the network being partially observed.

### 4.2 Recovering misspecified networks with a latent variable

**Fig. 5:**
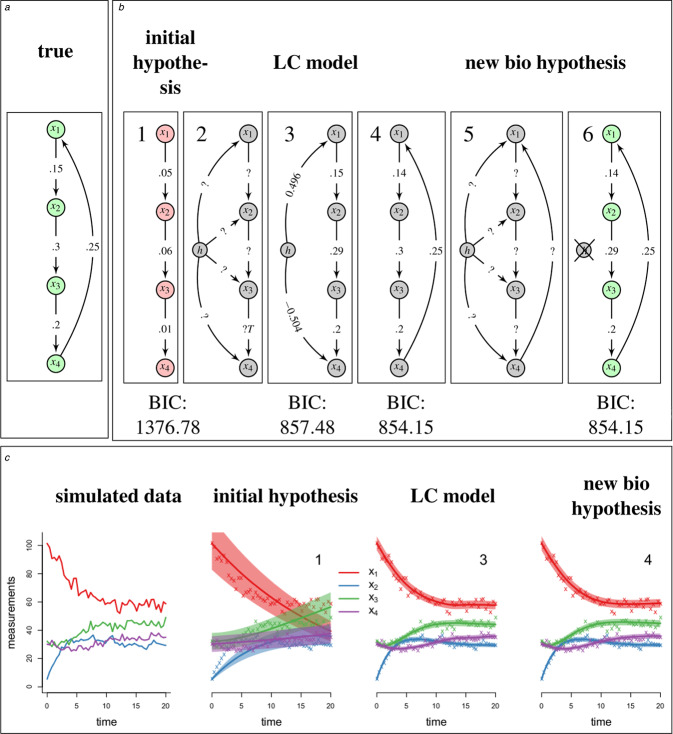
True course of hidden influence *a* True network from which the data is simulated *b* Schematic representation of the workflow (1) Misspecified network (2), (3) LC model suggests a feedback loop (4)–(6) Model with feedback loop cannot be further improved *c* Corresponding data and model fits

Our method can be used for a guided repair of a wrongly specified network. We demonstrate this using artificial data in a further example. In this example, the network from which we simulate time‐dependent observations consist of four players that are connected with each other in a forward cascade ending with a feedback loop, as shown in Fig. 5*a*. However, we assume that the initial hypothesis suggests a network structure with a missing feedback loop. Furthermore, we do not assume known reaction rates ***k***; thus, we incorporate the fitting of ***k*** into the application of our method.

Fig. 5 shows that we can simultaneously reconstruct the misspecified network structure and estimate ***k*** very well. The model without a feedback loop is best estimated with parameters $\hat{k}=(0.05,0.06,0.01){\;}^{T}$ and has a BIC value of 1376.78 [Fig. 5*b*(1)]. The identified LC has a positive interaction with the first species *x*_1_(*t*) and a negative interaction with the last species *x*_4_(*t*). This suggests that a feedback loop might be missing in the network specification. The corresponding BIC value is 857.48. The estimate $\hat{k}=(0.15,0.29,0.20){\;}^{T}$ is close to the true ***k*** [Fig. 5*b*(2,3)]. The constellation of interactions between the hidden component and ***x*** suggests a feedback loop. Inclusion of this loop further improves the BIC value to 854.15 and slightly alters $\hat{k}=(0.14,0.30,0.20){\;}^{T}$ [Fig. 5*b*(4)]. Subsequent application of our method does not identify a LC which significantly improves the model fit [Fig. 5*b*(5, 6)].

This example demonstrates the ability of our method to recover misspecified network structures. We repeated the presented example with random data 100 times and concluded the same missing feedback in 97% of the repetitions (results not shown). However, in general networks, misspecifications may occur in very a complex manner; thus, overall it will be difficult to always apply our method under all conditions. Nevertheless, even if the network structure cannot be recovered completely, a hidden component may indicate which network components are candidates for refining the network structure and whether inhibition or activation of certain network components is more likely to improve a given model.

### 4.3 JAK2‐STAT5 signalling pathway

The simulation study in Section 4.1 has shown that our estimation procedure can reliably detect and quantify a hidden influence on a given network. We now focus on models and real‐world data from the literature. A prominent and well‐studied example is the erythropoietin (Epo) signalling pathway which tranduces Epo stimulation via JAK2‐STAT5 [32]. Epo signalling plays an important role in proliferation, differentiation and survival of erythroid progenitor cells [33]. After binding of the Epo hormone to its receptor, STAT5 can also bind. Subsequently, dimerisation of STAT5 results in a translocation to the nucleus where the STAT5 dimer acts as a transcription factor.

Several models exist which explain the molecular dynamics in various ways [34, 35, 36, 37]. We analyse immunoblotting data which have already been analysed by a basic model [34] using the following system of ODEs

(21) $$\begin{array}{rl} {\dot{x}}_{1} & =-k_{1}x_{1}{\text{EpoR}}_{A}\\ {\dot{x}}_{2} & =-k_{2}x_{2}^{2}+k_{1}x_{1}{\text{EpoR}}_{A}\\ {\dot{x}}_{3} & =-k_{3}x_{3}+0.5k_{2}x_{2}^{2}\\ {\dot{x}}_{4} & =+k_{3}x_{3} \end{array}$$

Here, the different states of STAT5 are cytoplasmic unphosphorylated STAT5 (denoted by *x*_1_), cytoplasmic phosphorylated monomeric STAT5 (*x*_2_), cytoplasmic phosphorylated dimeric STAT5 (*x*_3_) and STAT5 in the nucleus (*x*_4_). EpoR_A_ describes the Epo‐induced tyrosine phosphorylation which can be measured up to a scaling factor. The initial values are *x*_1_(0) > 0 (to be estimated) and *x*_2_(0) = *x*_3_(0) = *x*_4_(0) = 0.

In the above mentioned literature, the model given in (21) is further refined by, for example, introducing an additional transition from nuclear STAT5 to the cytoplasmic unphosphorylated state, thus completing the loop from *x*_1_ to *x*_4_, or introducing time delays. These model refinements typically lead to an improved representation of the measured data, confirmed by, for example, likelihood ratio tests, information criteria (AIC/BIC) or Bayes factors. To start from the best‐known model, we extend the refined model by incorporating a hidden influence. As a first step, we consider

(22) $$\begin{array}{rl} {\dot{x}}_{1} & =-k_{1}x_{1}{\text{EpoR}}_{A}+a_{1}h\\ {\dot{x}}_{2} & =-k_{2}x_{2}^{2}+k_{1}x_{1}{\text{EpoR}}_{A}+a_{2}h\\ {\dot{x}}_{3} & =-k_{3}x_{3}+0.5k_{2}x_{2}^{2}+a_{3}h \end{array}$$

Here, we do not consider the fourth row of (22) because we have no information about *x*_4_ as we use the measurements of experiment number 1 provided as supporting material in [34]. These measurements describe the total amount of cytoplasmic tyrosine phosphorylated STAT5, that is, *y*_1_ = *k*_5_(*x*_2_ + 2*x*_3_), the total amount of cytoplasmic STAT5, *y*_2_ = *k*_6_(*x*_1_ + *x*_2_ + 2*x*_3_), and the Epo‐induced tyrosine phosphorylation, *y*_3_ = *k*_7_EpoR_A_. All three measured time‐varying variables were experimentally quantified up to scaling factors denoted by *k*_5_, *k*_6_ and *k*_7_. Evidently, only transformations of the ODE components *x*_1_, …, *x*_3_ are observed. Furthermore, a system comprising only *y*_1_, *y*_2_ and *y*_3_ cannot be described in closed form. For that reason, we also include the auxiliary variable *x*_3_. The differential equations for the observed and LCs are as follows

(23) $$\begin{array}{rl} {\dot{y}}_{1} & =\frac{k_{1}k_{5}y_{2}y_{3}}{k_{6}k_{7}}-\frac{k_{1}y_{1}y_{3}}{k_{7}}-2k_{3}k_{5}x_{3}+k_{5}(a_{2}+2a_{3})h\\ {\dot{y}}_{2} & =-2k_{3}k_{6}x_{3}+k_{6}(a_{1}+a_{2}+2a_{3})h\\ {\dot{x}}_{3} & =-k_{3}x_{3}+\frac{k_{2}y_{1}^{2}}{2k_{5}^{2}}-\frac{2k_{2}y_{1}x_{3}}{k_{5}}+2k_{2}x_{3}^{2}+a_{3}h \end{array}$$

We further refine the model by completing the loop from *x*_4_ to *x*_1_ and including a time delay, as has been done previously [38]. The authors suggested the use of a linear chain trick [39] and introduced a delayed loop. Thus, two (or possibly more) additional variables in the system of differential equations are introduced

(24) $$\begin{array}{rl} {\dot{x}}_{1} & =-k_{1}x_{1}{\text{EpoR}}_{A}+2k_{4}z_{2}+a_{1}h\\ {\dot{x}}_{2} & =-k_{2}x_{2}^{2}+k_{1}x_{1}{\text{EpoR}}_{A}+a_{2}h\\ {\dot{x}}_{3} & =-k_{3}x_{3}+0.5k_{2}x_{2}^{2}+a_{3}h\\ {\dot{x}}_{4} & =+k_{3}x_{3}-k_{4}z_{2}\\ {\dot{z}}_{1} & =\frac{1}{\tau }(x_{3}-z_{1})\\ {\dot{z}}_{2} & =\frac{2}{\tau }(z_{1}-z_{2}) \end{array}$$

Analogously, we can transform these equations to counterparts depending on *y*_1_, *y*_2_ and *y*_3_

(25) $$\begin{array}{rl} {\dot{y}}_{1} & =\frac{k_{1}k_{5}y_{2}y_{3}}{k_{6}k_{7}}-\frac{k_{1}y_{1}y_{3}}{k_{7}}-2k_{3}k_{5}x_{3}+k_{5}(a_{2}+2a_{3})h\\ {\dot{y}}_{2} & =-2k_{3}k_{6}x_{3}+2k_{4}k_{6}z_{2}+k_{6}(a_{1}+a_{2}+2a_{3})h\\ {\dot{x}}_{3} & =-k_{3}x_{3}+\frac{k_{2}y_{1}^{2}}{2k_{5}^{2}}-\frac{2k_{2}y_{1}x_{3}}{k_{5}}+2k_{2}x_{3}^{2}+a_{3}h\\ {\dot{z}}_{1} & =\frac{1}{\tau }(x_{3}-z_{1})\\ {\dot{z}}_{2} & =\frac{2}{\tau }(z_{1}-z_{2}) \end{array}$$

This representation captures the dynamics of the observed variables. The right‐hand side of (25) depends on the observed components *y*_1_ to *y*_3_, the hidden component *h*, the unobserved component *x*_3_ and the two artificially introduced delay variables *z*_1_ and *z*_2_. For this reason, we must estimate *x*_3_, *z*_1_ and *z*_2_ prior to *h*. This is achieved by numerically computing the solution of the model given in (24) without considering the hidden component (i.e. ***a*** = **0**) and using the approximations for *x*_3_, *z*_1_ and *z*_2_ arising from this model. Once these quantities are determined, we estimate the three transformed weighting coefficients $\tilde{a}=(k_{5}(a_{2}+2a_{3}),k_{6}(a_{1}+a_{2}+a_{3}),a_{3})$ and use the estimates for *x*_3_, *z*_1_ and *z*_2_ as input in the new iteration. This procedure is repeated until convergence. Once $\tilde{a}$ is successfully obtained, we simply calculate ***a*** from $\tilde{a}$ up to the scaling factors *k*_5_ and *k*_6_.

**Fig. 6:**
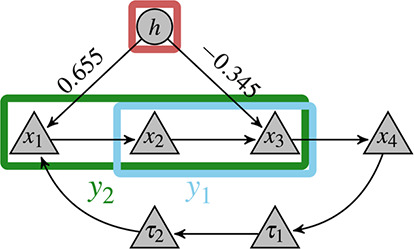
Schematic representation of the JAK2‐STAT5 signalling pathway Four different states of STAT5 are regulated by a LC *h* with different weights, as estimated by our method. Observed variables *y*_1_ and *y*_2_ are linear combinations of the single states *x*_1_ to *x*_3_ *τ*_1_ and *τ*_2_ represent artificial delay variables

Fig. 6 shows a schematic description of the estimated model given in (25). According to our estimation performed by best subset selection, the hidden component interacts only with the first and third states of STAT5. Interestingly, the interaction direction (activating *x*_1_ and inhibiting *x*_3_) hints at a translocation of STAT5 from its nuclear state to the cytoplasm as also hypothesised by other authors [34].

**Fig. 7:**
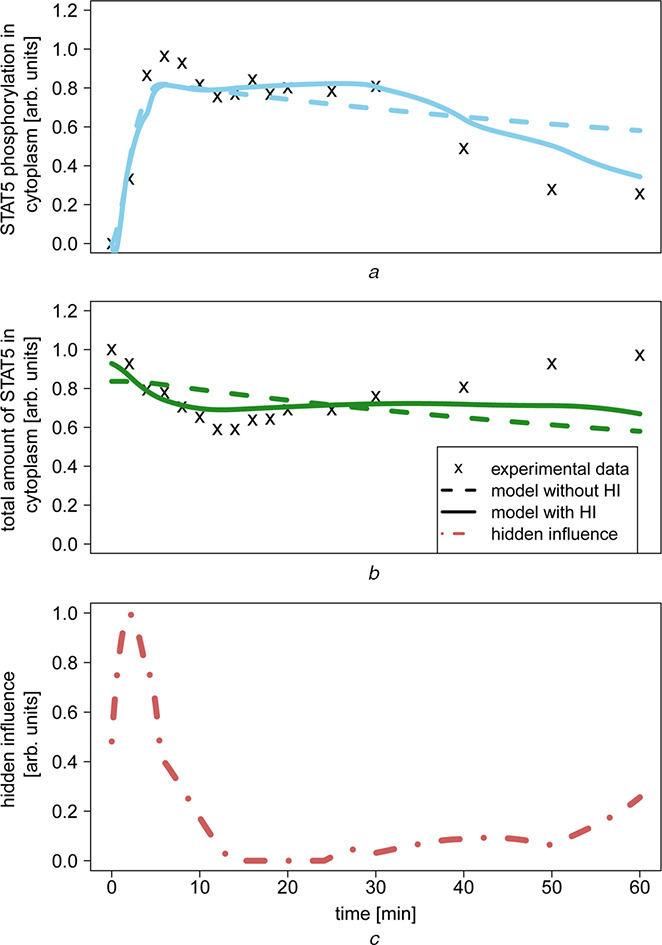
Experimental data and the estimated time courses of y_1_ and y_2_ *a* Experimental data and model fitting for STAT5 phosphorylation in cytoplasm (*y*_1_) *b* Experimental data and model fitting for the total amount of STAT5 in cytoplasm (*y*_2_) Model that includes no hidden component is indicated by the dashed lines, whereas that which includes a hidden component is indicated by solid lines Model with a hidden influence produces a time course that better fits the experimental data *c* Estimated time course of the hidden component, which exhibits a strong peak at the beginning of the experiment, quickly drops to 0 and begins to increase again after 30 min

Figs. 7*a* and *b* show the experimental data and the estimated time courses of *y*_1_ and *y*_2_. The model with a hidden component *h* outperforms the model without *h* because it best represents the experimental data. Most importantly, the time course produced with a hidden component is considerably more flexible but does not overfit the data. The time course of the estimated hidden component (third panel of Fig. 7*c*) exhibits large values at the beginning of the experiment, decreases and then begins increasing after 30 min. Our interpretation of this behaviour is that an external quantity should be present at the beginning of the experiment (or shortly after); thus, the entire signalling pathway is kick‐started. This external stimulus depletes completely and its influence slowly begins increasing after 30 min, bringing the entire system into equilibrium with the inhibition of the dimerised STAT5, and simultaneously the activation of the monomeric STAT5.

## 5 Conclusion

The main objective of this study is to provide a new method for model extension by introducing a hidden component to known networks. With the proposed method, we cannot only derive the relative time course of the hidden component but also predict the influence of the hidden component on all other network components.

We first fit splines to the observed components or to observation functions that are affine linear transformations of the former. The advantage of splines is that we can immediately compute the corresponding time derivative. On the basis of the observation error distribution, we apply maximum‐likelihood estimation and model selection. By so doing, we can estimate a combination of the time course of a hidden component and the weights that lead to the best model in terms of data faithfulness without overfitting.

The method was applied to artificial data to test robustness and applicability. The results suggest a robust and good performance for the identification of the time course of the hidden component even in scenarios with high noise intensity.

One application of our method is the detection of misspecified networks. As a demonstration, we chose a network which included a feedback loop that was missing in the model specification. The loop was successfully recovered, thus providing a promising application variant of our technique. Our method, however, is not a tool for general network inference in its current form. An automation of the process by combining theory from network topology estimation with the proposed latent variable model presents a possible extension in the future work.

We applied the method to the well‐studied JAK2‐STAT5 signalling pathway. Model extension with a LC was performed on a system of ODEs with introduced delay. Our method could improve the model quality in terms of BIC and produced results which are in conformity with other methods suggested in the literature.

A key element in general model building is the estimation of parameters and possibly topology from data. Here we propose to interpret model estimation as a latent variable problem in a dynamical system. We target applications in which latent variables are influencing observations but not vice versa. A coupling in *h*(*t*) in the sense of feedback of observed network components to the LC is possible; however, we mainly see two limitations of this approach. First, additional assumptions about *h*(*t*) must be made, and second, including *h*(*t*) into the system of ODEs limits its shape and does not allow for additional flexibility.

We currently only allow linear model extensions. In ongoing work, we extend our method to more general settings. More specifically, we consider multiple independent latent variables that influence a system of differential equations. In this scenario, in addition to the estimation of the latent time courses, we study possible ways of separation of the single variables. Modelling endogenous dependencies between latent and system variables without losing the flexibility of the hidden quantity is another extension that may be addressed in the future. Furthermore, although the proposed method was extensively tested in designed simulation scenarios, many additional challenges such as dependent errors, low sample sizes (in the sense of a very short time‐series or a large amount of missing data) and non‐linear ODEs remain, and at the same time, present additional method extensions. Finally, one might investigate additional model selection methods that are applicable to our method and produce stable and efficient results. Examples are established methods such as likelihood ratio tests, lasso [40] and elastic net [41]. Extending the method to Bayesian theory would further allow the application of Bayes factors [42] and thermodynamic integration [43, 44].

For the method presented here, we intentionally chose to separate the two major estimations into two steps, and both steps can be associated with two major modelling perspectives [3]. While fitting the spline parameters can be associated with a statistical perspective, exploiting the network structure for inference of the latent time‐course and its interaction weights is closely connected to the mathematical modelling perspective. Model selection and thus network prediction, as discussed in Section 3.4, bring the method back to the statistical perspective. Formulating the problem as a joint optimisation of all parameters involved (reaction rates, spline parameters and noise parameters) is possible. This, however, leads to a considerably more complex and computationally intensive method.

As we demonstrate in Appendix 2, the performance of our method depends on the quality of the spline approximation. This quality will typically suffer if the modelled data are sparse, contain extreme outliers, are corrupted by a high amount of noise or the chosen spline representation cannot resemble fluctuations of the observed time‐series appropriately.

The results of the proposed method can be employed as a promising aid for guiding future experiments, thus helping to complete the systems biology loop [45, 46] between experimental data and model analysis.

## Appendix 8

### 8.1 Appendix 1: Log‐normally distributed multiplicative noise

Normally distributed error terms, as in (10), imply that the noise level is independent of the magnitude of the measurements. Although this assumption is commonly made, it is not appropriate for all biological applications, for example, when concentrations are measured over time. This assumption is particularly problematic for concentrations close to zero because the model description of $x_{i}^{\text{obs}}(t_{j})$ can then easily become negative. This difficulty is avoided by assuming multiplicative log‐normally distributed noise

$$x_{i}^{\text{obs}}(t_{j})=x_{i}(t_{j})\cdot \varepsilon_{ij},\;\varepsilon_{ij}\overset{\text{iid}}{∼}LN\left(-\frac{\sigma^{2}}{2},\sigma^{2}\right)$$

The parameter choice of the log‐normal distribution is motivated because of the implication $E\left(x_{i}^{\text{obs}}(t_{j})|x_{i}(t_{j})\right)=x_{i}(t_{j})$.

The distribution of *ε_ij_* immediately propagates to the measurements

$$x_{i}^{\text{obs}}(t_{j})|x_{i}(t_{j})\overset{\text{iid}}{∼}LN\left(\log(x_{i}(t_{j}))-\frac{\sigma^{2}}{2},\;\sigma^{2}\right)$$

As discussed in Section 3.2, we exploit the ODE structure of our system which leads to the approximate distribution

$$x_{i}^{\text{obs}}(t_{j})|{\hat{x}}_{i}^{\text{ode},a}(t_{j})\overset{\text{iid}}{∼}LN\left(\log({\hat{x}}_{i}^{\text{ode},a}(t_{j}))-\frac{\sigma^{2}}{2},\;\sigma^{2}\right)$$

The parameter *σ* ^2^ is estimated as

$${\hat{\sigma }}_{\text{ML}}^{2}=-2+2\sqrt{1+\frac{\sum_{i=1}^{N}\sum_{\;j=0}^{n}{\left[\log\left(\left(x_{i}^{\text{obs}}(t_{j})\right)\left({\hat{x}}_{i}^{\text{ode},a}(t_{j})\right)\right)\right]}^{2}}{N(n+1)}}$$

The diagonal elements of the expected Fisher information matrix are derived as

$$I_{k}(a,\sigma^{2})=\left\{\begin{array}{cc} \frac{1}{\sigma^{2}}\sum_{i}\sum_{j}{\left(\frac{\left(\partial /\partial a_{k}){\hat{x}}_{i}^{\text{ode},a}(t_{j}\right)}{{\hat{x}}_{i}^{\text{ode},a}(t_{j})}\right)}^{2} & k\leq N\\ \frac{N(n+1)}{2}\left(\frac{1}{\sigma^{4}}+\frac{1}{2\sigma^{2}}\right) & k=N+1 \end{array}\right.$$

### 8.2 Appendix 2: Example for parameter uncertainty calculation

In this section, we evaluate the goodness of fit for parameters associated with a specific example. For simplicity, we choose a small network comprising two species and normally distributed measurement noise. The two parameters of interest are *a*_1_ and *σ* ^2^. We analytically compute the CRLB for both parameters and examine whether it is achieved using a simulation with known parameters.

The specific network is described by (2) and Fig. 1*b* and we consider a network size *N* = 2 and linear combinations of the components. Without loss of generality, we assume *a*_2_ = 1 − *a*_1_ and *a*_1_ *∈* (0, 1).

The CRLB for parameter *a*_1_ involves $\left(\partial /\partial a_{1}){\hat{x}}_{i}^{\text{ode},a}(t_{j}\right)$. The value of this term depends on the numerical method chosen for solving the ODE. If the Euler method is used, the recursive solution has the form

$$\begin{array}{rl} {\hat{x}}_{1}^{a}(t_{\;j+1}) & ={\hat{x}}_{1}^{a}(t_{j})+\Delta (c_{1}{\hat{x}}_{1}^{a}(t_{j})+c_{2}{\hat{x}}_{2}^{a}(t_{j})+a_{1}{\hat{h}}^{a}(t_{j}))\\ {\hat{x}}_{2}^{a}(t_{\;j+1}) & ={\hat{x}}_{2}^{a}(t_{j})+\Delta (c_{3}{\hat{x}}_{2}^{a}(t_{j})+c_{4}{\hat{x}}_{1}^{a}(t_{j})+(1-a_{1}){\hat{h}}^{a}(t_{j})) \end{array}$$

with *c*_1_ = *− k*_2_ − *k*_1_, *c*_2_ = *k*_3_, *c*_3_ = *− k*_4_ − *k*_3_, *c*_4_ = *k*_2_ and Δ denoting the time step, and ${\hat{x}}_{i}^{a}$ being a shortened version of ${\hat{x}}_{i}^{a,\text{ode}}$. We assume that the initial values *x_i_* (*t*_0_) do not depend on *a*_1_. One can then show by full induction that, for *j* = 1, …, *n*

(26)
$$\begin{array}{rl} \frac{\partial x_{1}(t_{j})}{\partial a_{1}} & =\frac{F_{1}(t_{j})}{(1-a_{1}){\;}^{2}}-\frac{F_{2}(t_{j})}{a_{1}^{2}}\\ \frac{\partial x_{2}(t_{j})}{\partial a_{1}} & =\frac{F_{3}(t_{j})}{a_{1}^{2}}+\frac{F_{4}(t_{j})}{(1-a_{1}){\;}^{2}} \end{array}$$

with

$$F(t_{\;j+1})=F(t_{j})+\Delta \left(\begin{array}{c} c_{1}F_{1}(t_{j})+c_{2}F_{4}(t_{j})+\frac{1}{2}{\hat{h}}_{2}^{0}(t_{j})\\ c_{1}F_{2}(t_{j})-c_{2}F_{3}(t_{j})\\ c_{3}F_{3}(t_{j})-c_{4}F_{2}(t_{j})-\frac{1}{2}{\hat{h}}_{1}^{0}(t_{j})\\ c_{3}F_{4}(t_{j})+c_{4}F_{1}(t_{j}) \end{array}\right)$$

and ***F*** (*t*_0_) = (0, 0, 0, 0)^T^. ${\hat{h}}_{i}^{0}(t)$ are the unweighted estimates of the time course of the hidden component, as described in (7). The entries of ***F*** (*t_j_*) are independent of *a*_1_. For given *σ* ^2^, ***k***, *a*_1_, spline approximations of ***x*** (*t*) and data dimensions, (26) allows the analytical computation of the expected Fisher information *I*(*a*_1_), and hence that of the CRLB *I^−^* ^1^(*a*_1_). For this specific example, the expected Fisher information matrix has a diagonal form and *I^−^* ^1^(*σ* ^2^) equals $\frac{2\sigma^{4}}{N(n+1)}$.

The just derived CRLBs are lower bounds for the MSE of the maximum‐likelihood estimates. Our estimation procedure, however, consists of two steps: spline approximation and maximum‐likelihood estimation, each entailing uncertainty in the parameter estimates. We hence computed Monte Carlo estimates for the MSE of *a*_1_ and *σ* ^2^ using two different approaches. First, we used the true hidden time course during the estimation procedure. The resulting empirical MSE is the one resulting from the maximum‐likelihood step and is bounded below by *I^−^* ^1^. Second, we estimated the hidden time course as well. The resulting MSE is slightly larger and accounts for the uncertainty of the overall estimation procedure.

The results of the simulation are shown in Table 1. We examine different combinations of *σ* ^2^ and ***a***. For each combination, we simulate 500 time courses at 100 time points and estimate the parameters. We numerically compute the MSE of these 500 estimates. In the table, we show this MSE and the corresponding CRLB for a given parameter combination.

**Table 1:** Results of the parameter uncertainty simulation

MSE (CRLB)			
		$a=\left(\begin{array}{c} 0.5\\ 0.5 \end{array}\right)$	$a=\left(\begin{array}{c} 0.9\\ 0.1 \end{array}\right)$
maximum‐likelihood approximation			
*σ* = 1	*a*_1_	9.90 × 10*^−^* ^6^ (1.63 × 10*^−^* ^6^)	3.73 × 10*^−^* ^9^ (9.27 × 10*^−^* ^10^)
	*σ* ^2^	1.07 × 10*^−^* ^2^ (1.00 × 10*^−^* ^2^)	1.02 × 10*^−^* ^2^ (1.00 × 10*^−^* ^2^)
*σ* = 0.5	*a*_1_	2.33 × 10*^−^* ^6^ (4.08 × 10*^−^* ^7^)	9.04 × 10*^−^* ^10^ (2.32 × 10*^−^* ^10^)
	*σ* ^2^	6.19 × 10*^−^* ^4^ (6.25 × 10*^−^* ^4^)	6.38 × 10*^−^* ^4^ (6.25 × 10*^−^* ^4^)
*σ* = 0.1	*a*_1_	9.06 × 10*^−^* ^8^ (1.62 × 10*^−^* ^8^)	3.54 × 10*^−^* ^11^ (9.27 × 10*^−^* ^12^)
	*σ* ^2^	1.04 × 10*^−^* ^6^ (1.00 × 10*^−^* ^6^)	9.83 × 10*^−^* ^7^ (1.00 × 10*^−^* ^6^)
maximum‐likelihood and spline approximations			
*σ* = 1	*a*_1_	1.55 × 10*^−^* ^4^ (1.24 × 10*^−^* ^5^)	5.23 × 10*^−^* ^6^ (1.13 × 10*^−^* ^7^)
	*σ* ^2^	7.59 × 10*^−^* ^2^ (1.00 × 10*^−^* ^2^)	1.33 × 10*^−^* ^1^ (1.00 × 10*^−^* ^2^)
*σ* = 0.5	*a*_1_	1.62 × 10*^−^* ^5^ (3.13 × 10*^−^* ^6^)	9.57 × 10*^−^* ^7^ (3.44 × 10*^−^* ^8^)
	*σ* ^2^	4.29 × 10*^−^* ^3^ (6.25 × 10*^−^* ^4^)	5.44 × 10*^−^* ^3^ (6.25 × 10*^−^* ^4^)
*σ* = 0.1	*a*_1_	6.22 × 10*^−^* ^7^ (1.25 × 10*^−^* ^7^)	2.69 × 10*^−^* ^8^ (1.08 × 10*^−^* ^9^)
	*σ* ^2^	6.24 × 10*^−^* ^6^ (1.00 × 10*^−^* ^6^)	4.31 × 10*^−^* ^6^ (1.00 × 10*^−^* ^6^)

MSE and corresponding CRLB (in parentheses) for the two parameters of interest.

The results of Table 1 show that the CRLB is achieved for *σ* ^2^ if we consider only maximum‐likelihood approximation. If we additionally consider the uncertainty introduced by the spline approximation, the ratio between MSE and CRLB increases slightly. For *a*_1_, we observe a similar result in that the ratio increases if we consider spline approximation, while, nevertheless, providing MSE values that are very close to the corresponding CRLB. Another interesting result is the increase in fit quality for smaller *σ* ^2^ values, as we already discussed in Section 3.3.
